# Disorders/Differences of Sex Development Presenting in the Newborn With 46,XY Karyotype

**DOI:** 10.3389/fped.2021.627281

**Published:** 2021-04-22

**Authors:** Silvano Bertelloni, Nina Tyutyusheva, Margherita Valiani, Franco D'Alberton, Fulvia Baldinotti, Maria Adelaide Caligo, Giampiero I. Baroncelli, Diego G. Peroni

**Affiliations:** ^1^Paediatric and Adolescent Endocrinology, Division of Pediatrics, Department of Clinical and Experimental Medicine, University of Pisa, Pisa, Italy; ^2^Clinical Psychologist, Bologna, Italy; ^3^Laboratory of Molecular Genetics, Azienda Ospedaliero Universitaria Pisana, Pisa, Italy

**Keywords:** 46, XY disorder of sex development, testis, fetal gonadal hormones, ambigous genitalia, sex assignment

## Abstract

Differences/disorders of sex development (DSD) are a heterogeneous group of congenital conditions, resulting in discordance between an individual's sex chromosomes, gonads, and/or anatomic sex. The management of a newborn with suspected 46,XY DSD remains challenging. Newborns with 46,XY DSD may present with several phenotypes ranging from babies with atypical genitalia or girls with inguinal herniae to boys with micropenis and cryptorchidism. A mismatch between prenatal karyotype and female phenotype is an increasing reason for presentation. Gender assignment should be avoided prior to expert evaluation and possibly until molecular diagnosis. The classic diagnostic approach is time and cost-consuming. Today, a different approach may be considered. The first line of investigations must exclude rare life-threatening diseases related to salt wasting crises. Then, the new genetic tests should be performed, yielding increased diagnostic performance. Focused imaging or endocrine studies should be performed on the basis of genetic results in order to reduce repeated and invasive investigations for a small baby. The challenge for health professionals will lie in integrating specific genetic information with better defined clinical and endocrine phenotypes and in terms of long-term evolution. Such advances will permit optimization of counseling of parents and sex assignment. In this regard, society has significantly changed its attitude to the acceptance and expansion beyond strict binary male and female sexes, at least in some countries or cultures. These management advances should result in better personalized care and better long-term quality of life of babies born with 46,XY DSD.

Si sta come

d'autunno

sugli alberi

le foglie

G. Ungaretti (1918)

## Introduction

**Figure 1:**
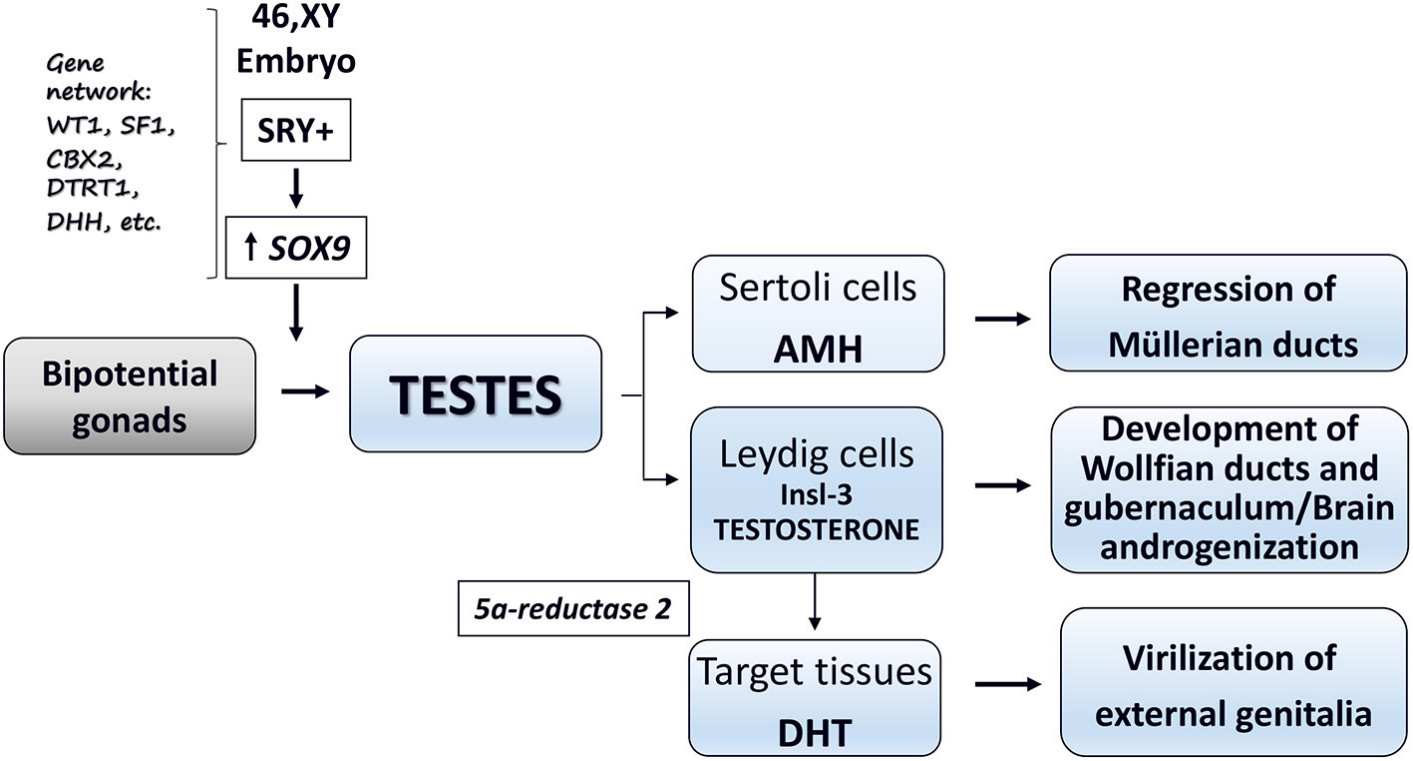
Schematic representation of male sex differentiation *in utero*.

Phenotypic sex is the result of a coordinated and sequential series of fetal events controlled by complex gene systems, transcription factors, and optimal hormone secretion during critical developmental windows (1–4). Sex development starts at fertilization by the establishment of chromosomal sex (XX or XY). In human fetuses with XY karyotype, the *SRY* (*sex determining region on the Y chromosome*) and the related gene network promote the formation of functional testes (sex determination). The final step (sex differentiation) leads to the formation of the phenotypic sex (i.e., development and stabilization of the external and internal genitalia as well as the programming of the male or female brain and reproductive axis). In the 46,XY fetus, this step is based on the hormones secreted by primordial testes and peripheral response of target tissues to these hormones (1–4) (Figure 1).

Disorders (or differences) of sex development (DSD) are defined as congenital conditions which feature an alteration in the development of genetic, gonadal, or phenotypic sex (5, 6). This terminology recognizes the simple but fundamental pathway of the nature of sex chromosomes (XX or XY) organizing the development of the gonads (testis or ovary) whose hormones (effectively anti-Müllerian hormone and androgens in fetal life) determine the genital phenotype (male or female) (7). The 46,XY DSD group includes a wide spectrum of conditions due to genetic variants, altered hormonal secretion, or abnormal peripheral sensitivity to testicular hormones that are able to change the usual male fetal development, causing varying degrees of under-virilization (5–7). 46,XY DSD may be divided into two broad categories: (1) disorders of sex determination characterized by abnormal gonadal development; (2) disorders of sex differentiation characterized by altered production of testicular hormones or altered peripheral response to steroid or protein hormones produced by the fetal testis (Table 1).

**Table 1:** Main forms of 46,XY DSD (4, mod).

**Disorders of gonadal (testis) development**
- Complete or partial gonadal dysgenesis (due to genetic variants in SRY, SOX9, NR5A1, WT1, DHH, DMRT1, etc.) - Ovotesticular DSD - Testis regression
**Disorders of androgen synthesis**
- LH receptor mutations - Smith-Lemli-Opitz syndrome - Steroidogenic acute regulatory protein mutations^*^ - Cholesterol side-chain cleavage (CYP1IA1)^*^ - 3β-hydroxysteroid dehydrogenase 2 (HSD3B2)^*^ - 17α-hydroxylase/17,20-lyase (CYP17)^*^ - P450 oxidoreductase (POR) - 17β-hydroxysteroid dehydrogenase (HSD17B3) - 5α-reductase2 (SRD5A2)
**Disorders of androgen action**
- Androgen insensitivity syndrome (complete, partial, minimal) - Drugs and environmental modulators of androgen receptor activity
**Disorders of AMH synthesis or action**
- Persistent Müllerian duct syndrome
**Other**
- Syndromic associations of male genital development (e.g. cloacal anomalies, Robinow, Aarskog, Hand-Foot-Genital, syndromes) - Vanishing testis syndrome - Isolated hypospadias (CXorf6) - Congenital hypogonadotropic hypogonadism - Cryptorchidism (INSL3, GREAT) - Environmental endocrine disruptors

* *Associated with congenital adrenal hyperplasia*.

The impact of 46,XY DSD in the life of the affected individuals and their families is immense, as these conditions require long-term clinical, endocrinological, and psychological management (5). Adequate management of the newborn with 46,XY DSD is challenging, because it affects sex assignment (and possible re-assignment), decisions on gonadal management (including oncological risk), hormone replacement therapy from adolescence onward (when needed), and lifelong health status (3, 5–8). Early correct diagnosis is a key factor for optimizing quality of life, but true diagnoses based on pathogenetic pathways is still not reached in some individuals (9–14), jeopardizing outcome.

In this paper, some aspects related to the diagnosis and management of newborns with 46,XY DSD are discussed, taking into consideration some personal views developed during years of clinical work and exchange of opinions with our colleague and friend Paolo Ghirri, a frontier soldier in the field of neonatal endocrinology.

## Clinical Presentation

Presentation of a newborn with 46,XY DSD may be characterized by varying degrees of ambiguity of genital phenotype, usually leading to easy identification during routine physical examination. In some instances, few clinical signs, such as mono- or bilateral inguinal herniae or mild hypospadias or micropenis associated with undescended testes, may be the only manifestations (4–6) (Table 2). Accurate phenotypic examination (appearance of the external genitalia, presence or absence of palpable gonads, measurement of the phallus or clitoral length, identification of the position of the urethral opening, presence or absence of a vagina or urogenital sinus) must be made (4, 6, 8, 15). A complete female phenotype or very mild undervirilization may delay the diagnosis for months or years [as may occur in complete and minimal androgen insensitivity syndrome (AIS) or complete gonadal dysgenesis]. Salt-losing crises due to adrenal insufficiency rarely occur in 46,XY DSD (Table 1) (5, 6, 8, 15). Valuable clinical scores were developed to grade the atypical genitalia (16, 17). Some well-written reviews or guidelines are available on how to perform the physical evaluation of neonatal genitalia (5, 6, 8, 18, 19). Readers are encouraged to refer to these for a detailed description, but neonatal phenotypes may be inconclusive for diagnosis in the absence of a clear family history (Table 2).

**Table 2:** Clinical findings of main 46,XY DSD (without adrenal insufficiency).

	**46,XY gonadal dysgenesis**	NR5A1 deficiency^*^	**Leydig cell hypoplasia**	**17β-HSD3 deficiency**	**5α-reductase 2 deficiency**	**Complete/partial/minimal Androgen resistance**
Prevalence	?	?	Very rare	1: 147.000°	? °	1: 20/90.000
Inheritance	Variable	AD	AR	AR	AR	X-linked
Gene	*SRY, DHH*, etc.	*NR5A1*	*LHR*	*17β-HSD3*	*SRD5A2*	*AR*
Chromosome	variable	9q33.3	2p21	9q22	2p23	Xq11–12
External genitalia	Female	Female to male	Female to ambiguous	Female to ambiguous	Female to ambiguous	Female to ambiguous to male
Wolffian structures	No	Variable	No	variable	Yes	No (complete) to variable to male (minimal)
Müllerian structures	Yes	Variable	No	No	No	No
Gonads	Streak	Testes	Testes	Testes	Testes	Testes
Puberty	No	No/virilization	No	Virilization	Virilization	Femminilizatio to virilization
Sex change	No	Sometimes	No	30–50%	~75%	No (complete/minimal) to sometimes (partial)

*AD, autosomal dominant; AR, autosomal recessive*.

* *No adrenal insufficiency in heterozygous state; adrenal insufficiency is operative in homozygous state*.

° *Frequent in some specific populations with a high rate of consanguineous marriage*.

Prenatal diagnosis may occur due to the appearance of atypical genitalia on prenatal ultrasound or a mismatch between phenotype and genotype or a suggestive family history (20, 21). The growing use of prenatal genetic tests and high-resolution ultrasound is likely to increase the detection of fetuses with genotype/phenotype sex mismatch during pregnancy (22). The management of these conditions is a new challenge that requires expert counseling. Some genetic investigations could be performed prenatally, when possible. Complete evaluation should be performed after delivery to reach a correct diagnosis and to program personalized management. Prenatal diagnosis permits the opportunity for counseling and education of parents prior to the birth of a child with 46,XY DSD (8).

## Diagnostic Procedures

**Figure 2:**
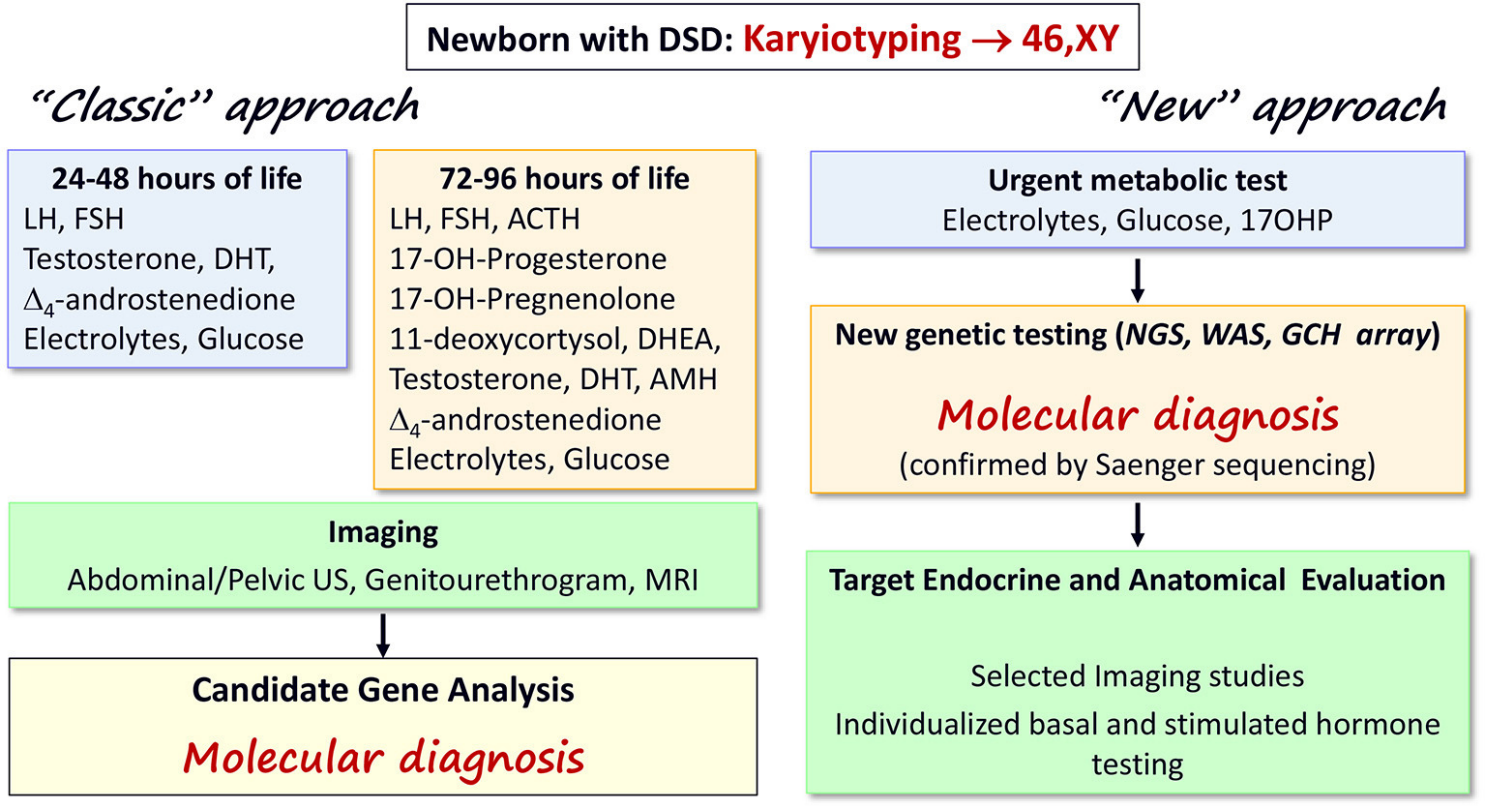
Diagnostic algorithms for early diagnosis of 46,XY DSD in the newborn. The “classic” (22) and the “new” approaches are summarized in the left and right panels, respectively. The “classic” scheme is based on serial assessment of several biochemical parameters and steroids during the first days of life as well as imaging studies to reach a suspected diagnosis that should be confirmed by gene analyses. The “new” scheme suggests that updated genetic testing may be performed as first line of investigation, leading to a molecular diagnosis. Then, only selected investigations are required reducing the stress for babies and their families of repeated (sometimes unnecessary) tests. The two schemes can work in parallel until more evidence is available; the availability of local resources must also be considered.

Rational investigations are mandatory in a newborn with 46,XY DSD to avoid repeated and invasive tests in a small baby (22). Balsamo et al. (19) proposed an extensive diagnostic scheme of laboratory assessment in the first 24–48 h of life (Figure 2, left panel). Such a scheme is still appropriate to avoid a salt-losing crisis, caused by rare forms of adrenal insufficiency (Table 1). During minipuberty (15–90 days after birth), hormonal status should be re-evaluated (8). This scheme is time and cost consuming and it may not result in a specific diagnosis because of the difficulties in steroid determination and because testicular protein hormone assays are unavailable in some clinical laboratories and countries (8, 23). Therefore, a parallel approach should be considered (24) (Figure 2, right panel). This approach suggests the use of advanced genetic technologies (i.e., next generation sequencing, whole exome sequencing, targeted CGH array) as the first-line test after karyotyping (24) which may result in a molecular diagnosis. After a genetic diagnosis, selected investigations should be performed to detail the clinical and biochemical phenotype, minimizing unnecessary tests, sampling, and analyses. The molecular diagnosis will permit more rational sex assignment, recognizing the natural history of the identified 46,XY DSD (8, 18), the risk of gonadal neoplasia (25), the possibility for fertility (26, 27), and mental health (8). In addition, this approach may aid the understanding of the clinical and molecular characteristics of emerging DSD associated with oligogenic mutations, in which multiple hits may contribute to the phenotype (28). In our experience, patients presenting between 2007 and 2016 had a higher rate of correct diagnosis and reduced diagnostic delay in comparison with those presenting between 2000 and 2006. The advent of new genetic techniques strongly influenced this result (14).

A recent position paper not specific for DSD from European Reference Network on rare endocrine conditions (ENDO-ERN, www.endo-ern.eu) concluded that early diagnosis of a genetically based endocrine disorder contributes to precise management and helps the patients and their families in their self-determined planning of life (29). Furthermore, the identification of a causative genetic alteration allows an accurate prognosis of recurrence risks for family planning. Asymptomatic carriers of pathogenic variants can be identified, and prenatal testing might be offered, where appropriate (29). Pitfalls leading to potentially inconclusive results may be due to identification of variants of unknown significance and inconsistent associations between DSD phenotypes and molecular findings (8). Costs and availability of the new genetic technologies may be additional factors limiting their application in some clinical settings (8), which might be overcome by establishing centers of expertise at national levels or by international consortia.

## Sex Assignment

Sex assignment is one of the main issues in the management of a newborn with 46,XY DSD (8, 19).

In the past, the “optimal gender policy” hypothesis stated that gender identity was neutral at birth and developed in the postnatal period under the influence of social, familial, and cultural factors (30). According to this theory, if a child with a DSD is raised without gender and anatomical sex ambiguity, gender identity was expected to develop in line with assigned sex (30). This hypothesis determined the practice of early sex assignment; early genital surgery was consequently performed “to correct” the atypical genitalia according to assigned sex. Long-term studies showed that the “optimal gender policy” did not always lead to a satisfying adult quality of life and sexuality (31). In recent years, the management of 46,XY DSD has changed. New ideas on psycho-sexual development as the result of multifaceted genetic, hormonal, and psychosocial influences have arisen (3, 32–36). Both biological sex and psychosexual development are considered as a spectrum of possibilities rather than a simple binary male/female system (37, 38).

A more open approach is needed in babies where the sex may not be easily defined at birth and more time is needed in order to determine the natural inclination of an individual partly related to their prenatal hormonal milieu (33). For example, 46,XY individuals with *SRD5A2* deficiency assigned as female at birth showed high rates of sex change and gender dysphoria (56–63%) from adolescence onward (39, 40). Functional brain imaging studies of women with complete AIS in comparison with 46,XY male and 46,XX female controls suggest that testosterone modulates the microstructure of somatosensory and visual cortices and their axonal connections to the frontal cortex; testosterone may also influence functional connections from the amygdala (35). The high rate of gender role switch from female to male in some 46,XY DSD during puberty may be due to the prenatal brain androgenization from normal testosterone secretion during intrauterine life (41) (Table 2). In these individuals, different decisions at birth can determine different outcomes in adulthood (Table 3).

**Table 3:** 46,XY DSD: similar phenotypes, different decisions, different outcome in two people with 46,XY karyotype and atypical genitalia at birth.

**Person**	**Mary**	**Mario**
Description of phenotype at birth	Ambiguous genitalia with clitoromegaly, urogenital sinus, inguinal gonads	Ambiguous genitalia with severe proximal hypospadias, urogenital sinus, inguinal gonads
Assigned sex	Female	Male
Investigations	Repeated endocrine and imaging studies	Imaging study of genitalia
Early diagnosis	Gonadal dysgenesis in infant with Morris syndrome^*^	Male undervirilization
Procedures	Gonadal removal, feminizing surgery	Male reconstructive surgery
Adult outcome	Gender dysphoria (“I'm sick, I can't understand what I am”) plus other psychiatric disorders, social withdrawal, poor education level, and work opportunity	Married, satisfying social and sexual activity, spontaneous proven fertility (2 daughters), University degree, top positions in his work
Age at molecular diagnosis	30 years old	66 years old
Molecular diagnosis	Compound heterozygosity for *SRD5A2* gene variants	Compound heterozygosity for *SRD5A2* gene variants

*The same molecular diagnosis (compound heterozygosity for SRD5A2 gene variants) was made in adulthood*.

* *Complete androgen insensitivity does not present with ambiguous genitalia and functioning testes are present and therefore not “gonadal dysgenesis”*.

Although there is still an association between the external appearance of the genitalia and the choice of sex assignment, clear temporal trends pointing toward an increased likelihood of infants with 46, XY DSD being raised as boys has been reported (42). Some factors may explain the new tendency for male gender assignment in some 46,XY DSDs. Fertility potential has become an important issue to evaluate, because spontaneous or assisted paternity has been documented in some men with 46,XY DSD (26). Furthermore, new data showed that the gonadal cancer risk is relatively low in several forms (18, 25, 43). Thus, recommending early gonadectomy may not be necessary, since regular follow-up could be an adequate approach (41, 43). In addition, new RNA microarray technology is likely to lead to very early identification of gonadal neoplasia (44, 45). The past recommendation for female assignment based on easier surgery has been overcome by improvements in male reconstructive surgical techniques. However, some studies have reported that the majority of individuals with 46,XY DSD raised as females have not experienced gender dysphoria (46, 47). Thus, male gender assignment should not be the rule in every case.

Social and cultural factors may influence decisions on sex assignment and outcome (48). In some societies, female infertility precludes marriage, which also affects employment prospects and creates economic dependence. Religious and philosophical views may influence how parents respond to the birth of an infant affected by 46,XY DSD. There may be fatalism and guilt feelings related to congenital malformations or genetic conditions; poverty and illiteracy may impair access to health care or may preclude the availability of updated knowledge and new technologies (18, 19, 47, 48).

Because the long-term outcome of the early management of babies with 46,XY DSD remains largely based on evidence from small series or single reports, ethical guidelines for the management of infants with DSD must be taken into consideration (49). These state that the following principles should guide clinical decisions: minimizing physical and psycho-social risks, preserving the potential for fertility and satisfying sexual relations in adolescence and adulthood, leaving options open for the future if necessary, respecting the parents' wishes, beliefs and sociocultural tradition, when possible, to guarantee the best options for a healthy life (that is *a state of complete physical, mental and social well-being and not merely the absence of disease or infirmity; WHO, 1948*) (49). Future studies integrating genetic, endocrine, imaging, surgical, psychologic, and follow-up data will give more objective data to aid sex assignment.

## Multidisciplinary Teams

Each subject with 46,XY DSD should receive individualized care by an expert multidisciplinary team. This team should include medical specialists (pediatric endocrinologists, geneticists, reproductive medicine specialists, pediatric surgeons and urologists, mental health specialists, ethicists, etc.) as well as nurses, social workers, and patient associations to optimize family-centered care (5, 8, 15, 18). The teams should be available at reference centers clearly delineated in each country and they should work closely with smaller centers (hub and spoke model), because the birth of a baby with 46,XY DSD can occur in any neonatal unit. The multidisciplinary teams should collaborate in communicating the correct information on DSD to the parents as well as pros and cons of management. The multidisciplinary teams should help and support the anxieties of parents that may lead to premature and irreversible decisions (47). They should also share advanced knowledge (including by e-learning projects), diagnostic procedures, and facilities for patients. The team should include the laboratories performing analyses for DSD (19, 23) and should operate within a quality framework and actively engage in harmonization of diagnostic and management approaches, permitting sound comparable data. The DSD–Endo-ERN as well as the international Disorders of Sex Development (I-DSD) and International Congenital Adrenal Hyperplasia registry (I-CAH) registries are relevant examples of tools for improving practice by virtual expert networks, cooperation between expert healthcare centers, and multicenter research on rare disorders (50).

## Support Groups

Support groups may be invaluable to individuals with 46,XY DSD and their families (51, 52). They actively work to improve management and research in this field and to push healthcare systems toward higher standards of care (51). For couples expecting a baby with a genetic/phenotypic sex mismatch or parents of a newborn with 46,XY DSD, support groups may provide a context in which intimate issues of concern can be approached by sharing parents' and patients' experiences. Support groups can also help families find the best quality of care (*www.dsd.guidelines.org*; *www.dsd-life.eu*) (51, 52). Parents of a baby with 46,XY DSD should be encouraged to contact a dedicated support group to share emotions and information.

Concerns have been expressed about the authority of LGBT (lesbian, gay, bisexual, and trans) movements in representing the community of people with DSD, which might lead to misleading messages related to confusion in terminology or the clinical condition (53).

## Conclusions

The care of people with DSD quickly evolves as knowledge in this field accrues (4–8), but the birth of a baby with 46,XY DSD is still perceived as a “social” rather than a true “medical” emergency. Currently, there are no fully established or evidenced based “right” or “wrong” decisions in this difficult field, but every family has to find its own path with open support and objective information from expert teams when dealing with the specific nature of their child (27, 33, 54). While many patients fare well and have a good quality of life (46, 47, 55), other individuals have expressed uncertainty about belonging to a specific gender or have reported poor quality of life (38, 56) (Table 3). New knowledge, updated investigations, clear diagnoses, respect for newborns and their families, and improved collaboration among national and international networks are likely to result in better health of people with DSD. Support groups provide added value to help families and promote quality research and care. Qualified psycho-social care should be also planned to optimize lifelong quality of life. Improvements are needed on diagnostic schemes in the first days of life as well as objective criteria to assign sex, to predict the risk of germ cell cancers and unnecessary gonadal removal, and to optimize surgical procedures and future fertility options. We outline a different approach to the investigation of 46,XY DSD which involves genetic testing, following exclusion of a salt-losing crisis, as genetic testing advances and becomes more available in this area.

## Author Contributions

All authors conceived the paper, have written the first draft, revised, and approved the final paper.

## Conflict of Interest

The authors declare that the research was conducted in the absence of any commercial or financial relationships that could be construed as a potential conflict of interest.
